# The “3D Versascrew”: An Innovative Approach for Management of Bilateral Cleft Lip and Palate Case

**DOI:** 10.1155/2022/2635167

**Published:** 2022-10-27

**Authors:** M. V. Ashith, NS Suma, K. Mithun, Dhruv Ahuja

**Affiliations:** ^1^Department of Orthodontics and Dentofacial Orthopedics, Manipal College of Dental Sciences Mangalore(Manipal Academy of Higher Education Manipal), Mangalore, Karnataka, India; ^2^Department of Orthodontics, SVS Institute of Dental Sciences, Mahabubnagar, India; ^3^Department of Orthodontics, AJ Institute of Dental Sciences, Mangalore, Karnataka, India

## Abstract

The purpose behind the innovation of modified nasoalveolar moulding appliance was to achieve simultaneous anteroposterior and transverse correction in bilateral cleft cases with minimal vomeroid bending. This paper presents a 3D Versascrew appliance that has been used for presurgical nasoalveolar moulding in a 15-day-old neonate with bilateral cleft. Treatment was initiated at 30 days and continued for 90 days following which mild expansion of posterior maxilla with establishment of arch continuity and concomitant improvement in nasal morphology was achieved towards the end.

## 1. Background

The primary challenge in the management of bilateral cleft lip and palate disorder arises from the deficient columella and ectopic premaxilla [1]. The objective of nasoalveolar moulding in bilateral cleft cases is to reconstruct a symmetrically balanced lip and nose with good columellar length before surgery [2, 3]. Literature review shows different techniques by Grayson, Figueora, and Liou for presurgical management of the bilateral cleft palate [4]. Nevertheless, when rapid and large movements are performed in the patient, the vomer can be deformed, injured, and even fractured, especially at the prevomerine suture [1]. Another critical factor in presurgical nasoalveolar moulding is the timing of the treatment. This article presents the case report of a neonate with bilateral cleft treated by a 3D Versa appliance where presurgical nasoalveolar moulding was performed, the method of appliance fabrication and perusal of nasal outcome has been thoroughly explained. The innovative appliance used here is named 3D Versascrew as it consists of a 3D screw and after its versatility of correction in all 3 dimensions- transverse, vertical, and antero-posterior dimensions whereas, previous studies have shown correction with jack screw, which provides control in antero-posterior dimension [4].

## 2. Case Presentation

A 15-day-old male infant with bilateral cleft lip and palate was referred to our department. The parent's chief complaint was impaired facial appearance of the child and difficulty in feeding. The medical history of the child and parents was conventional.

**Figure 1:**
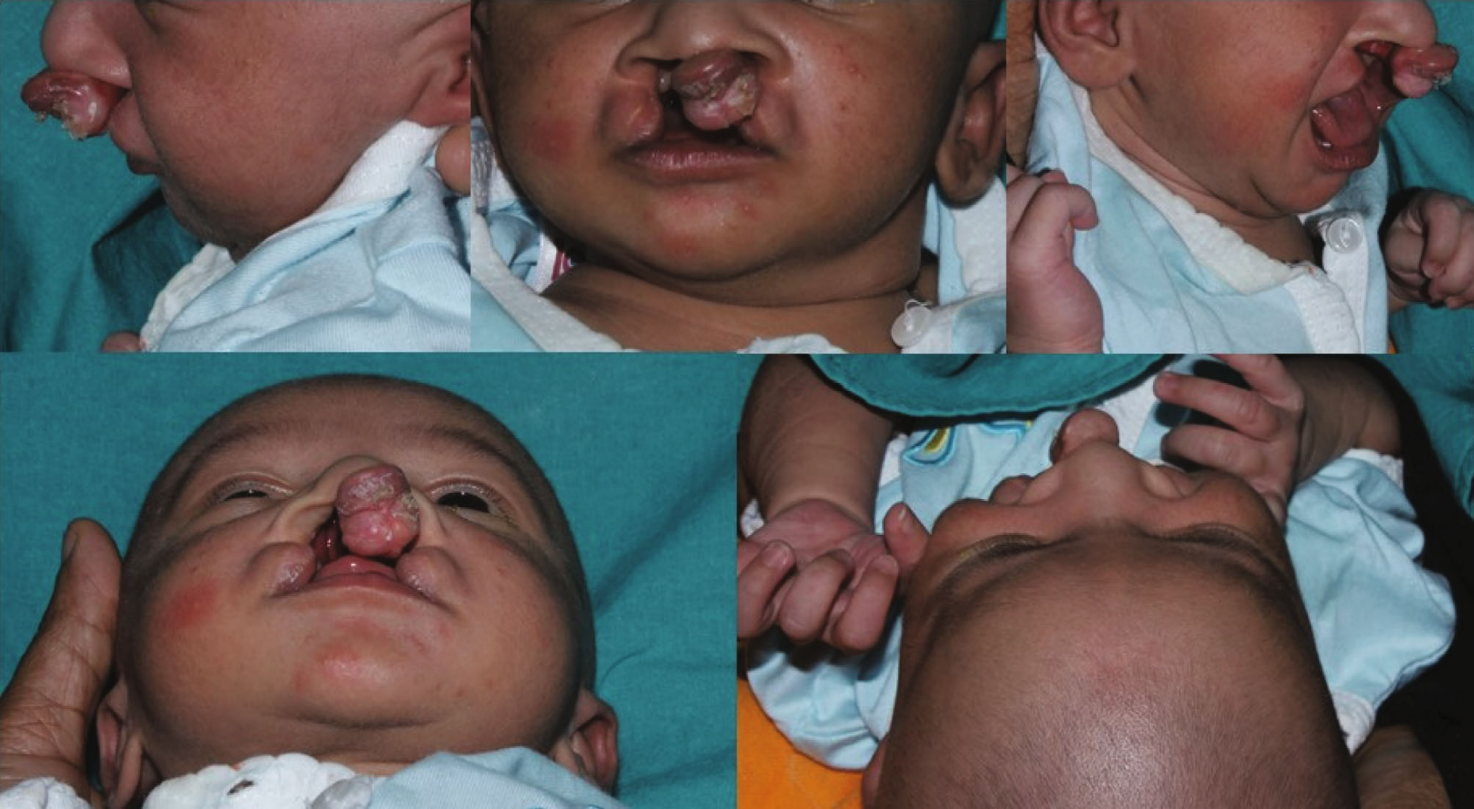
Pre-treatment: extraoral.

Extraoral and intraoral examination of the child revealed protruding and rotated premaxilla with the lip segments wide apart (Figure 1).

Depressed alar domes with reduced columella were other clinical features. Bird's eye view showed premaxilla almost obscuring the left nostril. After thorough clinical examination, the treatment method was explained to the parents and informed consent was obtained. The core intention of PNAM is to bring about correction of premaxillary position with minimal vomeroid bending, reduction in the cleft defect by approximation of segments, columellar lengthening, and improvement of nasal morphology. Minimal scarring is expected as the defect is minimized. The acrylic plate also functions as an obturator and aids in better feeding of the infant. As the patient reported difficulty in reporting for follow up appointments, we decided to treat the infant with a 3D Versascrew appliance, which is an active moulding plate incorporating a 3-way screw so that activations can be performed at home.

## 3. Treatment

**Figure 2:**
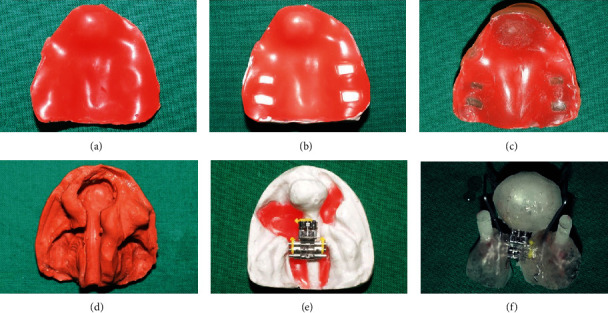
Steps in appliance fabrication (a) Primary wax impression. (b) Primary cast with wax spacer and tissue stops. (c) Special tray. (d) Polyvinylsiloxane impression. (e) Master cast with 3D screw in position. (f) 3D Versascrew appliance.

Primary impression of the infant's maxillary gumpad was made with moulding wax (Figure 2(a)). A special tray fabricated from the primary cast was used to make a definitive impression (Figure 2(b) and 2(c)). Impression of the upper gumpad was made using polyvinyl siloxane (Figure 2(d)) (REPROSILR, DENTSPLY) with the infant's head at a lower level than the rest of the body. A study model and a working model were poured using dental stone. Wax block outs were given to relieve the undercuts and alginate separating medium was deliberately applied all over the surface of the working model. 3D screw (Leone Company) was positioned slightly off-centred for premaxillary rotational correction with the anterior screw completely open to facilitate premaxillary retraction on activation. The screw position corresponded to the centre of the rotated predictive normal palate in the midst of posterior gum pad region (Figure 2(e)). Cold cure acrylic resin in dough stage was adapted uniformly over the cast surface and curing under pressure was done to ensure appliance with minimal porosity.

**Figure 3:**
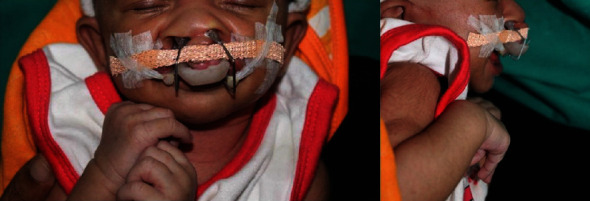
Insertion of a 3D Versascrew appliance.

Nasal stent wires were fabricated using TMA wire bent at almost right angle and further near the nasal bone the wire is bent at approximately at 80°. The ends of the wires were recurved to form a loop, which was covered with acrylic and coated with soft tissue liner to elevate the nasal dome. A thin layer of acrylic was then used to attach nasal stent wires to the appliance proper. These wires serve to initiate nasal correction from the very initial stages of treatment. Also, acrylic extensions to engage elastics were attached bilaterally to the appliance proper. The elastic was gently stretched and secured to cheek by means of a tape which served to prevent unseating of the appliance (Figure 2(f)). Finishing and polishing of the appliance were done to ensure a smooth surface and the appliance was placed in room temperature water overnight for leaching of excess monomer. Dynaplast (Johnson's) was used for active lip taping (Figure 3).

In order to prevent cheek irritation due to repeated active lip taping, Micropore tape (3 M) was placed on both the cheeks. This exerts a compressive force on the prolabium region, thus aiding in correction of premaxillary protrusion.

**Figure 4:**
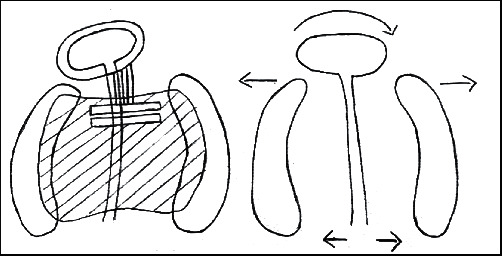
Off-centred screw position to correct the rotation of premaxilla.

The appliance was activated by closing the anterior screw and opening the posterior screw. Nasal stent wires were incorporated in the first visit to reduce the need for alteration of the appliance and to reduce the frequency of the visit. Activations were carried out only when cleft defect was ≤5 mm. The key to successful activation is the appearance of tissue blanching clinically. Biweekly activations were planned for the anterior screw to actively retract and centre the premaxilla. Posterior expansion was only minimally required to ensure the arch continuity; therefore, the posterior screw was activated only once a week. Prior to appliance insertion, the activation which is produced is 1–1.5 mm, which will not displace the nasal stent remarkably, the nasal stent wires are incorporated to elevate the nasal dome simultaneously the anterio-posterior screw activation will retract the premaxilla thus increasing the columella length and off-centred screw position to correct the deviation and rotation of the premaxilla (Figure 4). The gum pads were gently wiped to clear off the debris, and active lip taping was done using elastic adhesive bandage (Dynaplast). Customized activation protocol chart was given to the patient's parents and the method of activation and lip taping was demonstrated.

## 4. Outcome and Follow-up

By the end of active treatment, which on an average lasted about 2.5 months, we could achieve our treatment objectives with minimal vomeroid bending. The chief reason for this result was the anteroposterior rotation of premaxilla unlike pure retraction in conventional techniques. This also aided in deepening of the maxillary labial vestibule. The acrylic hood encasing the premaxilla passively depressed the premaxilla, which aided in establishing a good arch continuity.

**Figure 5:**
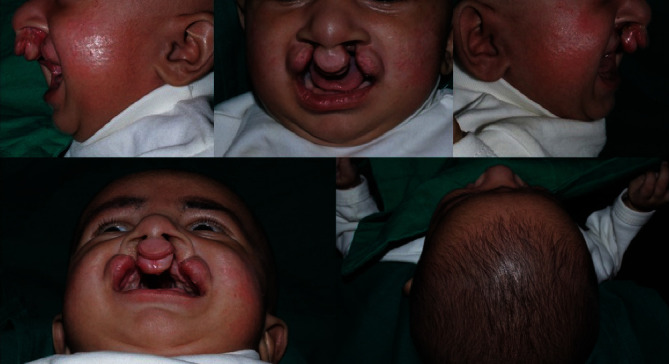
Post 3D Versascrew appliance: extraoral.

The treatment outcomes are listed herewith (Figure 5):

1. Correction of premaxillary position
2. Improved nasal contour with lengthening of columella
3. Deepening of the maxillary labial vestibule
4. Approximation of lip segments facilitating suturing without tissue tension
5. Appreciable reduction in cleft defect
6. Minimal vomeroid bending

**Figure 6:**
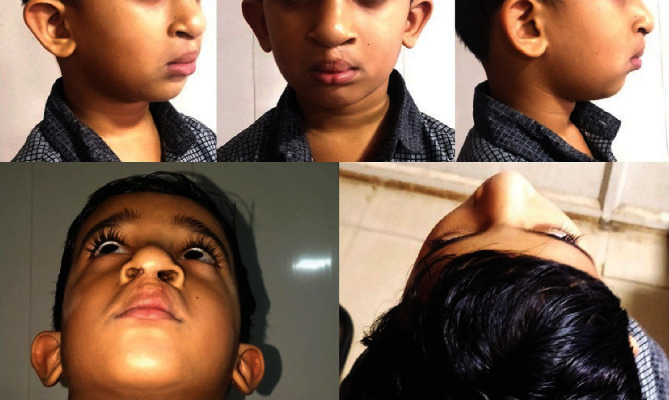
Four-year follow-up: extraoral.

The primary lip and palate repair was done at 6 months and 14 months, respectively. The approximation of the alveolar segments permits the surgeon to perform for better repair. A 4-year follow-up of the case shows the improvement in facial esthetics (Figure 6).

## 5. Discussion

Naso-alveolar moulding significantly improved the overall facial appearance of the infant by normalizing the premaxillary position, reduction of the alar base width, approximation of lip segments, columellar lengthening and increased nostril volume. The reduction in the cleft deformity and nasal correction prior to surgery makes lip closure less complicated and aids in better esthetics due to minimal scar tissue formation. Grayson stated that the plasticity of the cartilage faded over the first 6 months of age and a state of elasticity eventually set in [3]. In our case, as the patient presented at a very early age, we could make full advantage of plasticity of the cartilage and achieved good nasal moulding.

A study by Wojciech et al. [5] concluded that preoperative NAM in combination with primary gingivoperiosteoplasty reduces the need for secondary alveolar bone grafting by 60% in patients with unilateral cleft lip and palate. Similarly, in our study, naso-alveolar moulding achieved expansion of collapsed posterior segments which in turn, aided in accommodation of the retracted premaxilla and helped us achieve a good arch continuity. The permanent teeth were expected to erupt with a better periodontal support as the alveolar segments were aligned well and there were increased bony bridges across the cleft. In a study conducted by Severens et al. [6], it was found that the overall costs in cleft care were reduced by the lesser number of surgeries involved in the treatment. The same was achieved in our case with the decrease in the number of visits to the hospital and by achieving appreciable results in a short treatment duration.

A study by Neha et al. [4] advocated active retraction but posterior expansion was not performed whereas in our study, a 3D Versascrew appliance helped us achieve simultaneous premaxillary retraction and posterior maxillary expansion. Active retraction was preferred over passive in order to hasten the process of premaxillary retraction so that nasal moulding could be initiated at the earliest and also to facilitate at home activations. The activations were planned to keep the force levels gentle and to prevent any deleterious effects on the delicate vomer bone and maxillary segments. Furthermore, this is supported by a study done by the same authors, Neha et al., [7], where they have mentioned about the early correction of cleft with 3D expansion screw for simultaneously retraction and expansion using external nasal stents, whereas 3D Versacrew is a single appliance in which nasal stents are incorporated within the appliance for ease of use by the baby.

**Figure 7:**
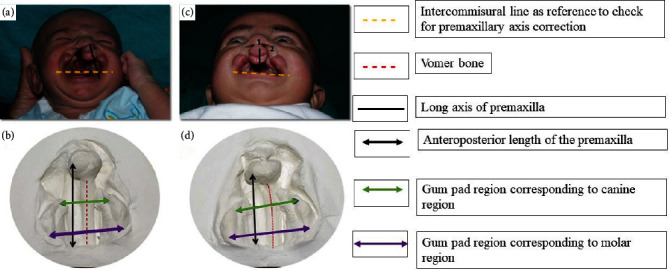
Comparison of pre- (a, b) and post-3D Versascrew appliance (c, d).

Construction of imaginary lines was done to quantify the correction achieved with the therapy. Imaginary line drawn connecting the most distal points of the posterior segments to mark the posterior extent of palate was labelled as posterior reference line. Premaxillary retraction was quantified by measuring the distance between the most prominent point on the superior aspect of the premaxilla and midpoint of posterior reference line (Figure 7).

Retraction of 4.5 mm was achieved in a period of 2.5 months. As controlled retraction was performed, there were minimal effects on the delicate vomer bone. An imaginary line drawn connecting the most mesial points of the posterior segments was labelled as anterior reference line and served as a guide to quantify the expansion measured at future canine region. Expansion of 2.5 mm was achieved in the mentioned period (Table 1). Appreciable reduction in the cleft defect was seen bilaterally minimizing the need for bone grafting in the future.

**Figure 8:**
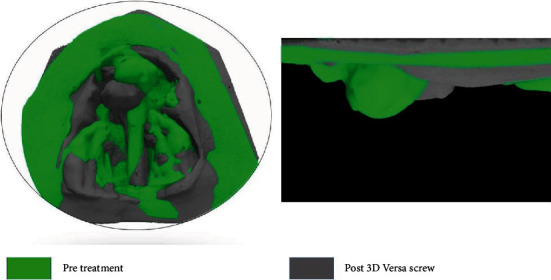
3D Superimposition of models.

The acrylic hood enclosing the premaxilla resulted in downward descent of the premaxilla and overcame the effect of premaxillary extrusion, while retraction, which was a concern with the previous techniques, and these changes can be appreciated in superimposition of pre- and post-treatment casts (Figure 8).

Thus, the “3D Versascrew appliance” results favourably in centering, retraction and passive depression of premaxilla with minimal vomeroid bending, modest posterior maxillary expansion with initiation of arch continuity and collateral refinement in nasal morphology and is indeed a versatile appliance true to its name.

## 6. Learning Points/Take Home Messages

1. Commencement of treatment of nasoalveolar moulding should be initiated at the earliest, preferably within 15 days of life
2. Nasoalveolar moulding decreases the number and cost of review-surgical procedures required for nasal defect rectification
3. 3D Versascrew appliance allows the growth of the alveolar processes in the right direction with minimal vomeroid bending, reshapes the flattened nose, and helps in minimizing the extent of complex surgeries

## Figures and Tables

**Table 1:** Comparative values of pre- and post-3D Versascrew appliance model analysis.

Parameter	Pre-treatment	Post-treatment
Antero-posterior length	38.5 mm	34 mm
Intercanine region of gumpad	21.5 mm	24 mm
Intermolar region of gumpad	33 mm	34.5 mm
